# Long noncoding RNA CCAT1 promotes hepatocellular carcinoma progression by functioning as let-7 sponge

**DOI:** 10.1186/s13046-015-0136-7

**Published:** 2015-02-19

**Authors:** Liang Deng, Shi-Bin Yang, Feng-Feng Xu, Ji-Hong Zhang

**Affiliations:** Department of Hepatobiliary Surgery, The Eastern Hospital of the First Affiliated Hospital, Sun Yat-sen University, Eastern Huangpu Road No. 183, Guangzhou, 510700 China; Department of Gastrointestinal and Pancreatic Surgery, The Eastern Hospital of the First Affiliated Hospital, Sun Yat-sen University, Eastern Huangpu Road No. 183, Guangzhou, 510700 China

**Keywords:** Long noncoding RNA, CCAT1, c-Myc, Hepatocellular carcinoma

## Abstract

**Background:**

Long noncoding RNAs (lncRNAs) have been identified as having functional roles in cancer biology and are deregulated in many cancers. The present study aimed to determine the expression, roles and functional mechanisms of a long noncoding RNA CCAT1 in the progression of hepatocellular carcinoma (HCC).

**Methods:**

CCAT1 expression levels in 66 pairs of HCC tissues and pair-matched noncancerous hepatic tissues were tested by real-time PCR. The effects of CCAT1 on HCC cells proliferation and migration were assessed using in vitro cell proliferation and migration assays. A computational screen of microRNAs (miRNAs) target sites in CCAT1 was conducted to search for specific miRNAs binding to CCAT1. The specific binding between CCAT1 and miRNAs was confirmed by RNA immunoprecipitation assay combined with luciferase reporter assay.

**Results:**

CCAT1 levels are markedly increased in HCC tissues compared with pair-matched noncancerous hepatic tissues. Up-regulation of CCAT1 is correlated with tumor size, microvascular invasion, AFP and poor prognosis. CCAT1 promotes the proliferation and migration of HCC cells. CCAT1 functions as a molecular sponge for let-7, antagonizes its functions, and leads to the de-repression of its endogenous targets HMGA2 and c-Myc. The effect of CCAT1 on HCC cell proliferation and migration is dependent upon its competitively binding to let-7.

**Conclusions:**

These data suggest that CCAT1 plays a pivotal role in HCC progression via functioning as let-7 sponge, and implicate the potential application of CCAT1 for the prognosis and treatment of HCC.

**Electronic supplementary material:**

The online version of this article (doi:10.1186/s13046-015-0136-7) contains supplementary material, which is available to authorized users.

## Introduction

Hepatocellular carcinoma (HCC) is the fifth most common solid tumor and the third leading cause of cancer-related deaths worldwide [1]. Most cases of HCC are attributed to chronic infection with either hepatitis B or C virus [2]. Unfortunately, unlike most malignancies, HCC patients remain have poor prognosis and high recurrence rate despite recent advances in surgical resection and medical treatment [3-5]. It is of paramount importance to understand the pathophysiological mechanisms contributing to HCC for developing new diagnosis and treatment strategies and improving the overall prognosis of HCC patients [6-8].

Long noncoding RNAs (lncRNAs), which are more than 200 nucleotides in length with limited protein coding potential, were recently identified as having functional roles in a variety of biological processes and disease states [9-13]. Notably, several lncRNAs are deregulated in many cancers, and the deregulation has been shown to result in aberrant gene expression that contributes to the progression of cancers, including HCC [14-17]. The overall pathophysiological contributions of lncRNAs to HCC remain largely unknown. Recently a novel long noncoding RNA colon cancer associated transcript-1 (CCAT1) was shown to be consistently upregulated in gastric carcinoma and colon cancer [18,19]. However, its expression, roles, and function mechanisms in HCC are still unknown and need to be investigated.

Recently, many RNA transcripts have been demonstrated to function as competing endogenous RNAs (ceRNA) by competitively binding common microRNAs (miRNAs) [20-22]. These ceRNAs generally share miRNA response elements with other transcripts targeted by that set of miRNAs. So these ceRNAs can function as sponges for that set of miRNAs, functionally preventing these targeted transcripts from being degraded [23,24]. Therefore we propose that whether CCAT1 also has roles as miRNAs sponges to modulate the functions of miRNAs.

In this study we found that CCAT1 is upregulated in human HCC tissues and is associated with poor prognosis. The upregulation of CCAT1 promotes the proliferation and migration of HCC cells through functioning as let-7 sponge.

## Materials and methods

### Patients

The 66 HCC tissues and their pair-matched noncancerous hepatic tissues utilized in this study were obtained with informed consent from patients who underwent radical resections at the Eastern Hospital of the First Affiliated Hospital, Sun Yat-sen University, Guangzhou, China. This study was performed with the approval of the Sun Yat-sen University Institutional Review Board.

### Cell culture

The human liver normal cell lines LO2 and QSG-7701, human HCC cell lines SMMC-7721, Hep3B, Huh7, and HepG2 were obtained from the Chinese Academy of Sciences Cell Bank. The cells were grown in Dulbecco’s modified Eagle’s medium supplemented with 10% fetal bovine serum (Gibco BRL, Gaithersburg, MD, USA), and were maintained in a humidified 37°C incubator with an atmosphere containing 5% CO2.

### RNA extraction and real-time PCR

Total RNAs was isolated using Trizol reagent (Takara, Dalian, China). First-strand cDNA was generated using the Reverse Transcription System Kit (Invitrogen, Carlsbad, CA, USA). Real-time PCR was performed using the standard SYBR Green PCR kit protocol in the StepOne Plus system (Applied Biosystems, Foster City, CA, USA). 18S rRNA was employed as an endogenous control for mRNA and lncRNA. The primer sequences used were as follows: for CCAT1, 5′-TTTATGCTTGAGCCTTGA-3′ (forward) and 5′-CTTGCCTGAAATACTTGC-3′ (reverse); for 18S rRNA, 5′-ACACGGACAGGATTGACAGA-3′ (forward) and 5′-GGACATCTAAGGGCATCACA-3′ (reverse); for HMGA2, 5′-TTCAGCCCAGGGACAACC-3′ (forward) and 5′-CTTTTGAGCTGCTTTAGAGGG-3′ (reverse); for c-Myc, 5′-GGGCTTTATCTAACTCGCTGTA-3′ (forward) and 5′-GCTATGGGCAAAGTTTCGTG-3′ (reverse). For miRNA analysis, real-time PCR was performed as above, using TaqMan miRNA assays according to the manufacturer’s instructions (Applied Biosystems). The real-time PCR reactions were performed in triplicate. The relative expression of RNAs was calculated using the comparative Ct method.

### Vectors construction

The complementary DNA encoding CCAT1 was PCR-amplified by the Pfu Ultra II Fusion HS DNA Polymerase (Stratagene, Agilent Technologies, Palo Alto, CA, USA) and subcloned into the pcDNA3.1 vector (Invitrogen). The primers used were 5′-CTAGCTAGCACAACATCGACTTTGAAGTT-3′ (forward) and 5′-CCCAAGCTTAAGACTTAATATACTTATATTTA-3′ (reverse). The pcDNA3.1-CCAT1 with point mutations in let-7 binding sites was synthesized by GenScript (Nanjing, China) and named pcDNA3.1-CCAT1-Mut. pSL-MS2-12X (Addgene) was double digested with BamH I and Xba I, and the MS2-12X fragment was subcloned into pcDNA3.1, pcDNA3.1-CCAT1 or pcDNA3.1-CCAT1-Mut, named pcDNA3.1-MS2, pcDNA3.1-MS2-CCAT1 or pcDNA3.1-MS2-CCAT1-Mut respectively. The let-7 binding region of either lncRNA-CCAT1 or lncRNA-CCAT1-Mut was amplified using PCR and subcloned into the pmirGLO vector (Promega, Madison, WI, USA) for Luciferase reporter assay. The primers used were 5′-CGAGCTCTCAACCCTGACGCTCTTTCTG-3′ (forward) and 5′-GCTCTAGATCACTCACCCACTGCTCACC-3′ (reverse).

### Transient transfection

Transfections were performed using the Lipofectamine 3000 kit (Invitrogen) according to the manufacturer’s instructions. let-7 miRNA mimics hsa-let-7a, hsa-let-7b, hsa-let-7c, and hsa-let-7e, miR negative control, anti-miR has-let-7b miRNA inhibitor and anti-miR control were purchased from Life Technologies (Ambion, Grand Island, NY, USA) and introduced into cells at a final concentration of 50 nM. The transfected cells were harvested at 48 hours after transfection.

### Construction of stable cell lines with overexpression or downregulation of CCAT1

To obtain cell lines stably expressing CCAT1, CCAT1-Mut, SMMC-7721 cells were transfected with the plasmid pcDNA3.1-CCAT1 or pcDNA3.1-CCAT1-Mut, and selected with neomycin (800 μg/ml) for four weeks. To obtain cell lines stably suppressing CCAT1, SMMC-7721 cells were transfected with the plasmid pGPU6/GFP/Neo-human-CCAT1 (designated shRNA-CCAT1) (GenePharma, Shanghai, China), and selected with neomycin (800 μg/ml) for four weeks. The shRNA-CCAT1 sequences used were as follows: 5′-CACCCCATTCCATTCATTTCTCTTTCCTATTCAAGAGATAGGAAAGAGAAATGAATGGAATGGTTTTTTG-3′ (forward) and 5′-GATCCAAAAAACCATTCCATTCATTTCTCTTTCCTATCTCTTGAATAGGAAAGAGAAATGAATGGAATGG-3′ (reverse).

### Cell proliferation assay

A total of approximately 5 × 10^3^ HCC cells were plated in 96-well plates. After 24 h of culture, cell proliferation was assessed using the Cell Counting Kit-8 (Dojindo, Tokyo, Japan) according to the manufacturer’s protocol. All experiments were performed in triplicate. The cell proliferation curves were plotted using the absorbance at each time point.

### Colony formation assay

One or two hundred cells were plated into 6-well plates and incubated in DMEM with 10% FBS at 37°C. Seven days later, the cells were fixed and stained with 0.1% crystal violet. The number of colonies, defined as >50 cells/colony, were counted.

### Transwell assay

Transwell assays were performed using poly-carbonate transwell filters (Corning, 8 μm) placed over the bottom chambers that were filled with culture medium containing 20% FBS. A sample of 4 × 104 cells was suspended in DMEM medium containing 1% FBS and seeded into the upper well. After 48 h, cells on the upper surface of the well were removed, and cells on the lower surface were fixed in paraformaldehyde and stained with 0.4% crystal violet. For each experiment, the number of transmigrated cells in five random fields on the underside of the filter was counted and photographed, and three independent filters were analyzed.

### RNA immunoprecipitation (RIP)

SMMC-7721 cells were co-transfected with pcDNA3.1-MS2, pcDNA3.1-MS2-CCAT1 or pcDNA3.1-MS2-CCAT1-Mut and pMS2-GFP (Addgene). After 48 hrs, cells were used to perform RIP experiments using a GFP antibody (Roche, Mannheim, Germany) and the Magna RIP™ RNA-Binding Protein Immunoprecipitation Kit (Millipore, Bedford, MA, USA) according to the manufacturer’s instructions.

### Luciferase reporter assay

pmirGLO, pmirGLO-CCAT1 or pmirGLO-CCAT1-Mut was co-transfected with let-7 mimics or miR NC into SMMC-7721 cells. The relative luciferase activity was normalized to Renilla luciferase activity 48 hr after transfection.

### Western blot analysis

Total cell lysates were prepared in a 1× sodium dodecyl sulfate buffer. Identical quantities of proteins were separated by sodium dodecyl sulfate-polyacrylamide gel electrophoresis and transferred onto nitrocellulose membranes. After incubation with antibodies specific for HMGA2, c-Myc (Cell Signaling Technology) or β-actin (Sigma-Aldrich, Saint Louis, MO, USA), the blots were incubated with IRdye 800-conjugated goat anti-rabbit IgG or IRdye 700-conjugated goat anti-mouse IgG and were detected using an Odyssey infrared scanner (Li-Cor, Lincoln, NE, USA).

### Immunohistochemical assay

Immunohistochemistry for the target molecules was performed on paraffin sections using a primary antibody against HMGA2 (Cell Signaling Technology, Boston, USA), and a horseradish peroxidase-conjugated IgG (1:500; Invitrogen), and the proteins in situ were visualized with 3, 3-diaminobenzidine. All slices were evaluated by two pathologists without knowledge of the clinical outcome. The staining intensities were evaluated in each sample and graded on a scale of 0 to 9.

### Statistical analysis

For comparisons, Student’s t-test, Wilcoxon signed-rank test, Pearson chi-square test, Log-rank test, Pearson correlation analysis, and Mann–Whitney U test were performed as indicated. All p values were two-sided and obtained using the SPSS 18.0 software package (SPSS, Chicago, IL, USA). Differences were defined as statistically significant for p-values < 0.05.

## Results

### CCAT1 is upregulated in human HCC tissues and is associated with poor prognosis

**Figure 1:**
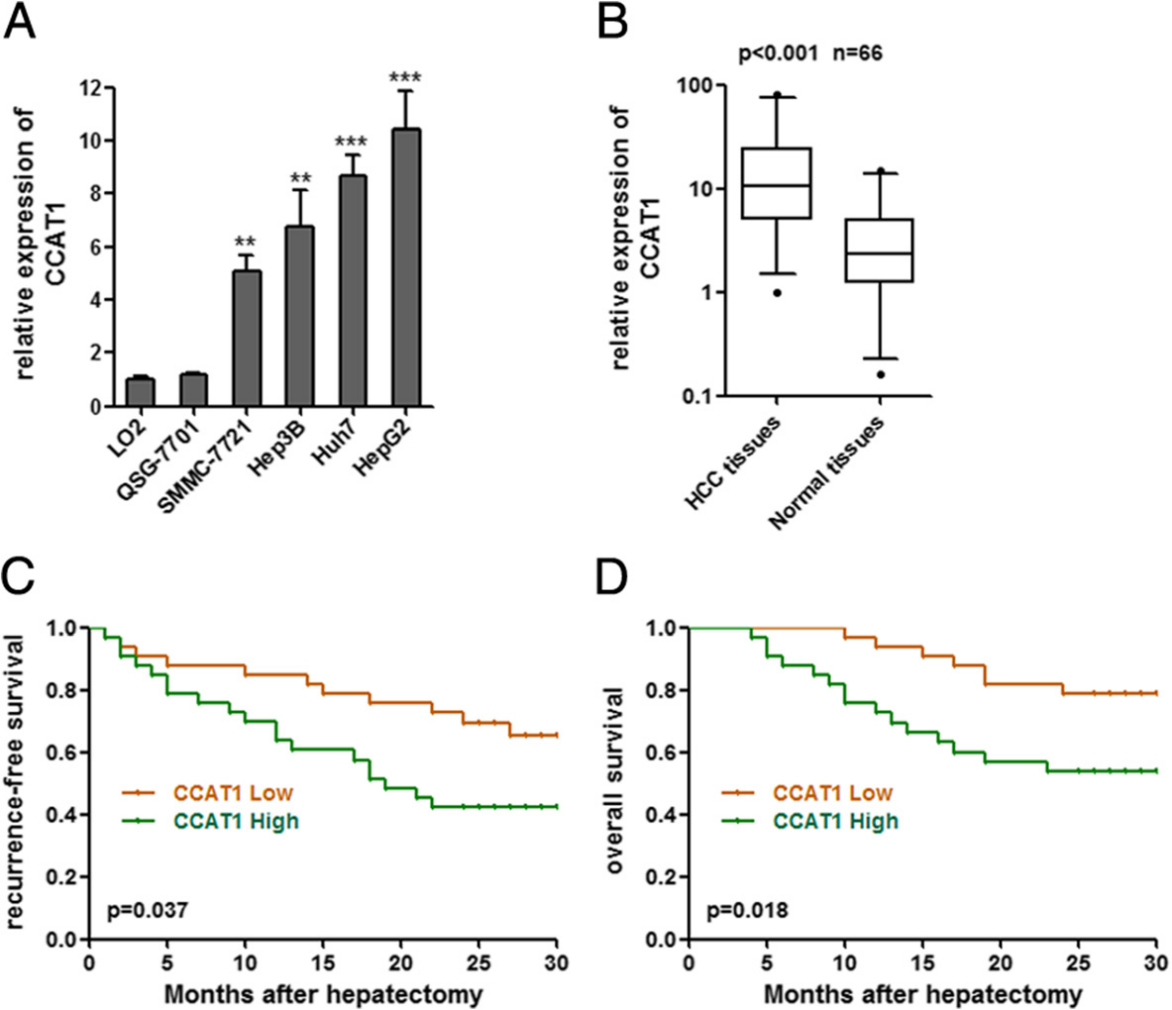
**CCAT1 expression in HCC and its association with patients’ prognosis. (A)** Expression of CCAT1 in four HCC cell lines and two liver normal cell lines. Data are presented as mean ± standard error based on at least three independent experiments. **p < 0.01, ***p < 0.001. **(B)** Differences in CCAT1 expression levels between HCC tissues and pair-matched noncancerous hepatic tissues. The expression of CCAT1 was normalized to that of 18S rRNA, which is an abundant and constitutively expressed non-coding RNA. Statistical differences between samples were analyzed using the Wilcoxon signed-rank test (n = 66, p < 0.001). **(C, D)** Kaplan–Meier analyses of correlations between the CCAT1 expression level and recurrence-free survival **(C)** or overall survival **(D)** of 66 HCC patients. The median expression level was used as the cut-off.

To explore the role of CCAT1 in HCC progression, we first examined the expression of CCAT1 in four human HCC cell lines (SMMC-7721, Hep3B, Huh7, and HepG2) and two liver normal cell lines (LO2 and QSG-7701). As shown in Figure 1A, elevated expression of CCAT1 was observed in all four HCC cell lines compared with that in liver normal cell lines. We then measured CCAT1 expression level in 66 pairs of HCC tissues and pair-matched noncancerous hepatic tissues. As presented in Figure 1B, the CCAT1 levels were significantly increased in HCC tissues as compared with pair-matched noncancerous hepatic tissues (p < 0.001 by Wilcoxon signed-rank test).

We next analyzed the correlation between CCAT1 expression levels and the clinicopathologic characteristics of the 66 HCC patients (Table 1). Correlation regression analysis showed that high expression of CCAT1 was significantly correlated with tumor size (p = 0.013), microvascular invasion (p = 0.032), and AFP (p = 0.011).

**Table 1:** **Association between CCAT1 expression and clinicopathological characteristics**

	**CCAT1**		**χ** ^**2**^	**p-value** ^*^
	**Low** ^**#**^	**High** ^**#**^		
All cases	33	33		
Age			0.306	0.580
>55	10	8		
≤55	23	25		
Gender			0.363	0.547
Male	25	27		
Female	8	6		
HBs antigen			0.183	0.669
Positive	29	31		
Negative	4	2		
AFP (ug/L)			6.418	0.011
>20	20	29		
≤20	13	4		
Tumor size (cm)			6.111	0.013
>5	10	20		
≤5	23	13		
Microvascular invasion			4.591	0.032
Present	6	15		
Absent	27	18		
Encapsulation			2.455	0.117
Complete	12	8		
No	21	25		

HBs antigen: hepatitis B surface antigen; AFP: alpha-fetoprotein.

^#^The median expression level was used as the cutoff. Low CCAT1 expression in each of the 66 patients was defined as a value below the 50th percentile. High CCAT1 expression in each of the 66 patients was defined as a value above the 50th percentile.

^*^For analysis of correlation between the expression levels of CCAT1 and clinical features, Pearson chi-square tests were used. Results were considered statistically significant at p < 0 .05.

We further examined whether the CCAT1 expression level was correlated with the outcome of HCC patients. Kaplan-Meier survival estimates showed that high CCAT1 expression in HCC tissues is significantly associated with worse recurrence-free survival (p = 0.037, log-rank test) and overall survival (p = 0.018, log-rank test) (Figure 1C, D). These results indicate that CCAT1 may play a pivotal role in the progression and malignant outcomes of HCC.

### Enforced expression of CCAT1 promotes the proliferation and migration of HCC cells

**Figure 2:**
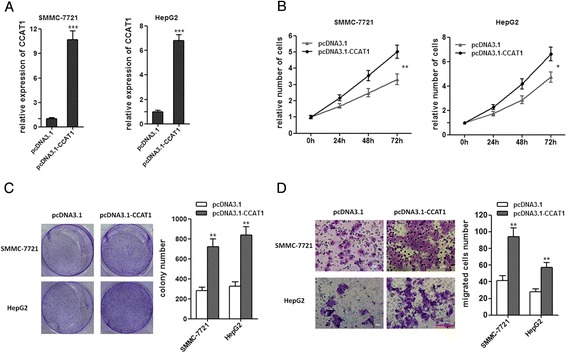
**Enforced expression of CCAT1 promotes the proliferation and migration of HCC cells. (A)** The expression of CCAT1 in SMMC-7721 and HepG2 cells with CCAT1 stably overexpressed. **(B)** CCAT1 promotes the proliferation of SMMC-7721 and HepG2 cells. Cell number was determined by the CCK-8 assay, and the relative number of cells to 0 h is presented. **(C)** The colony number of CCAT1 overexpressed SMMC-7721 and HepG2 cells per well in 6-well plates. **(D)** CCAT1 promotes cell migration by transwell assays. All values are presented as mean ± standard error based on at least three independent experiments. *p < 0.05, **p < 0.01, ***p < 0.001.

To investigate the biological functions of CCAT1 on HCC, we stably enhanced CCAT1 expression by transfecting CCAT1 expression vector (pcDNA3.1-CCAT1) into the HCC cell lines SMMC-7721 and HepG2, employing the pcDNA3.1 vector as a negative control (Figure 2A). Cell-counting kit 8 (CCK-8) assays indicated that enhanced expression of CCAT1 significantly promoted cell proliferation in SMMC-7721 and HepG2 cells (Figure 2B). Colony formation assays revealed that the CCAT1 transfected SMMC-7721 and HepG2 cells formed significantly more colonies than control SMMC-7721 and HepG2 cells (Figure 2C). These results suggest that CCAT1 plays an important role in regulating HCC cell proliferation. To evaluate the migration efficiency of HCC cells, transwell assays were performed. As presented in Figure 2D, enhanced expression of CCAT1 significantly increased cell migration in SMMC-7721 and HepG2 cells.

### Attenuated expression of CCAT1 inhibits the proliferation and migration of HCC cells

**Figure 3:**
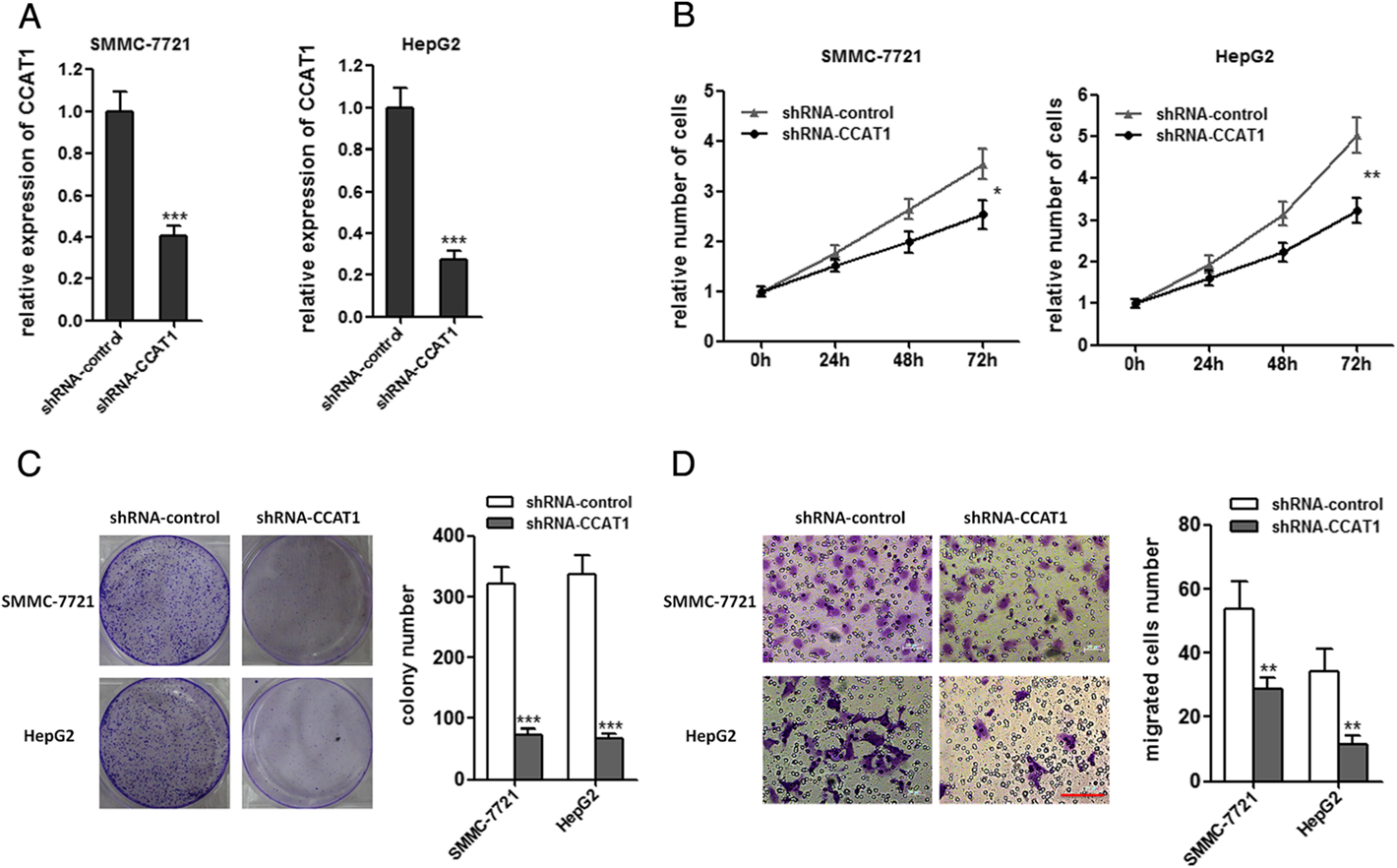
**Attenuated expression of CCAT1 inhibits the proliferation and migration of HCC cells. (A)** The expression of CCAT1 in SMMC-7721 and HepG2 cells with CCAT1 stably repressed. **(B)** Repression of CCAT1 decreases the proliferation of SMMC-7721 and HepG2 cells. Cell number was determined by the CCK-8 assay, and the relative number of cells to 0 h is presented. **(C)** The colony number of CCAT1 repressed SMMC-7721 and HepG2 cells per well in 6-well plates. **(D)** Repression of CCAT1 decreased cell migration by transwell assays. All values are presented as mean ± standard error based on at least three independent experiments. *p < 0.05, **p < 0.01, ***p < 0.001.

To further confirm the role of CCAT1 in HCC progression, we stably inhibited CCAT1 expression by transfecting CCAT1-specific shRNA into SMMC-7721 and HepG2 cells, using control shRNA as a negative control (Figure 3A). As demonstrated by CCK-8 assays, repression of CCAT1 significantly decreased cell proliferation in SMMC-7721 and HepG2 cells (Figure 3B). Colony formation assays revealed that CCAT1 repressed SMMC-7721 and HepG2 cells formed significantly less colonies than control SMMC-7721 and HepG2 cells (Figure 3C). As demonstrated by transwell assays, repression of CCAT1 decreased cell migration (Figure 3D). These data proved that CCAT1 play important roles in HCC progression.

### CCAT1 acts as a molecular sponge for let-7

**Figure 4:**
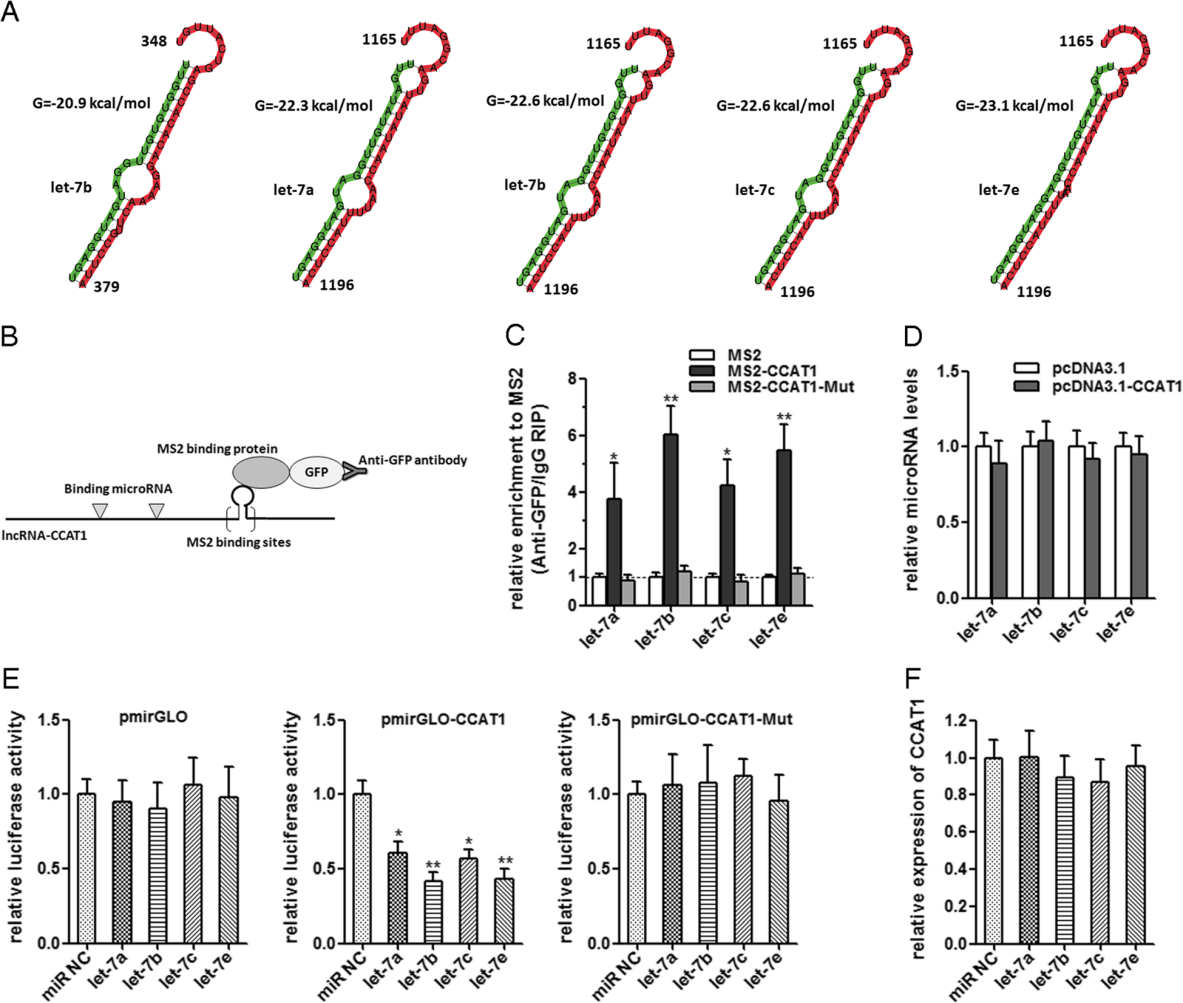
**CCAT1 acts as a molecular sponge for let-7. (A)** Bioinformatics predicted let-7 binding sites at two distinct positions in CCAT1. Partial sequences of CCAT1 and sequences of four let-7 subtypes are shown. Numbers are in nucleotides relative to the transcriptional start site of CCAT1. **(B)** Schematic outlining the MS2-RIP strategy to validate endogenous miRNA:CCAT1 binding. **(C)** MS2-RIP followed by miRNA qRT-PCR to detect miRNAs endogenously associated with CCAT1. **(D)** Expression levels of let-7 after the transfection of pcDNA3.1-CCAT1 or pcDNA3.1 into SMMC-7721 cells. **(E)** Luciferase activity in SMMC-7721 cells cotransfected with let-7 and luciferase reporters containing nothing, CCAT1 or mutant CCAT1. Data are presented as the relative ratio of firefly luciferase activity to renilla luciferase activity. **(F)** CCAT1 expression levels after the transfection of let-7 mimics or the miRNA negative control into SMMC-7721 cells. All values are presented as mean ± standard error based on at least three independent experiments. *p < 0.05, **p < 0.01.

Recently, many lncRNAs have been reported to function as sponges to bind specific miRNAs. To examine whether CCAT1 has a similar mechanism, prediction of miRNA target sites was performed by the online software MicroInspector (http://bioinfo.uni-plovdiv.bg/microinspector/) [25]. As shown in Figure 4A and Additional file 1: Table S1, CCAT1 contains two predicted let-7 targeting sites. It has been reported that let-7 inhibited tumor proliferation and migration, and induced apoptosis [26,27]. So we focused on let-7 as the primary candidate. To determine the direct binding between let-7 and CCAT1 at endogenous levels, we performed an RNA immunoprecipitation (RIP) to pull down endogenous miRNAs associated with CCAT1 and demonstrated via quantitative real-time PCR analysis that the CCAT1 RIP in SMMC-7721 cells was significantly enriched for let-7a, let-7b, let-7c, and let-7e compared to IgG, the empty vector (MS2), and CCAT1 with mutations in let-7 targeting sites (Figure 4B, C). In CCAT1-expressing cells, the levels of the four let-7 subtypes predicted to bind CCAT1 were not significantly changed (Figure 4D). These data demonstrated that CCAT1 bound let-7, but did not induce the degradation of let-7. For further confirmation, we constructed luciferase reporters containing CCAT1, which contains wild-type (WT) or mutated let-7 binding sites, for target investigates. We found that the let-7a, let-7b, let-7c, and let-7e mimics reduced the luciferase activities of the WT reporter vector but did not reduce the activity of the empty vector and mutant reporter vector (Figure 4E), supporting that let-7 are bona fide CCAT1-targeting miRNAs. Next, we speculated as to whether the let-7 could alter the expression of CCAT1. After transfection of let-7 mimics or the miRNA negative control into the SMMC-7721, we measured the expression of CCAT1. We found no significant difference in CCAT1 levels after transfection (Figure 4F). These data demonstrated that let-7 bound to CCAT1 but did not induce the degradation of CCAT1. All these data suggest that CCAT1 physically associated with let-7 and may function as a competing endogenous RNA for let-7.

### CCAT1 modulated expression of endogenous let-7 targets HMGA2 and c-Myc

**Figure 5:**
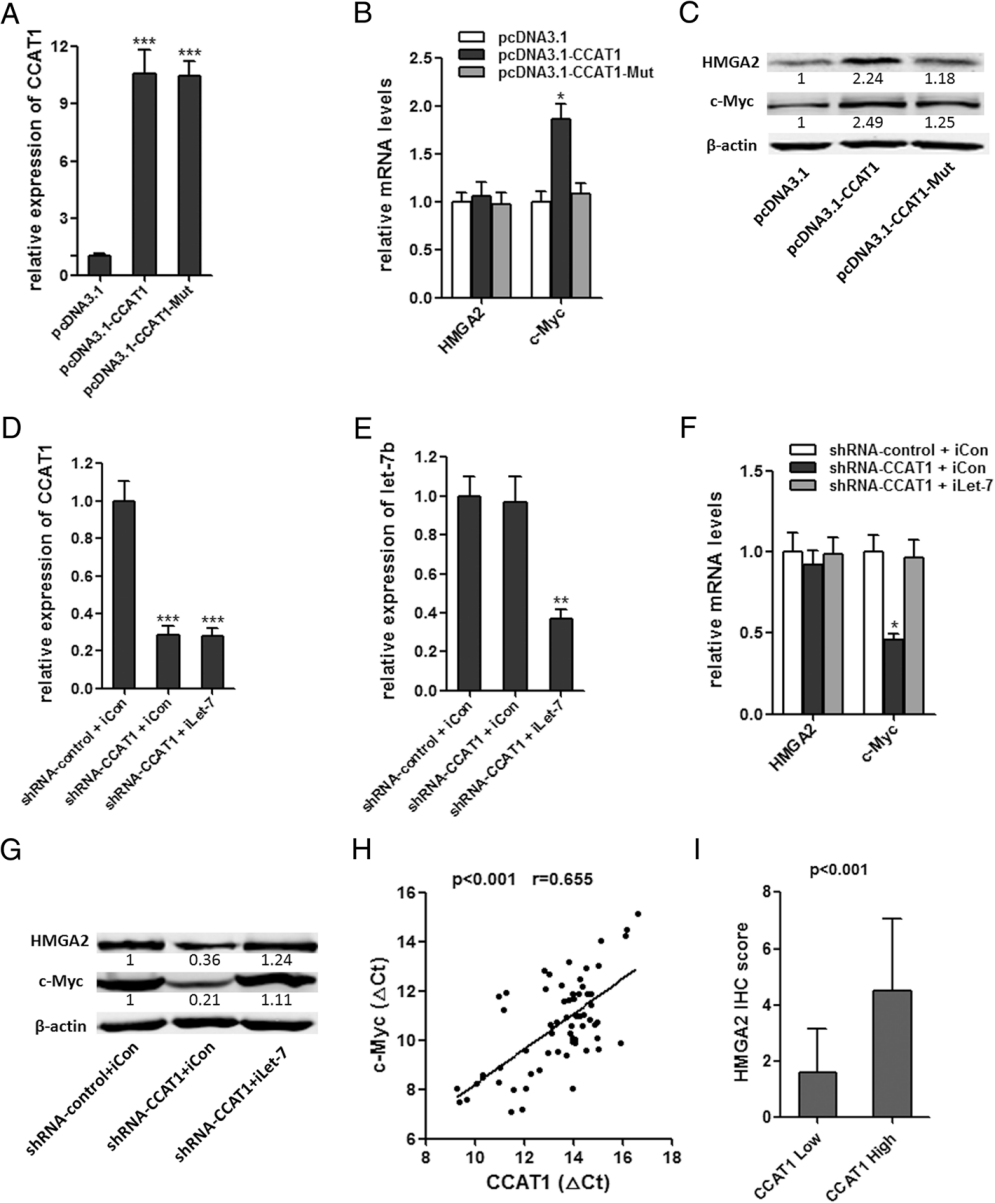
**Regulation of endogenous let-7 targets by CCAT1. (A)** The expression of CCAT1 in stable SMMC-7721 cell clones. **(B)** HMGA2 and c-Myc mRNA levels in stable SMMC-7721 cell clones. **(C)** HMGA2 and c-Myc protein levels in stable SMMC-7721 cell clones. **(D, E)** The expression of CCAT1**(D)** or let-7b **(E)** in indicated HepG2 cell clones after transfection of let-7 inhibitor. **(F)** HMGA2 and c-Myc mRNA levels in stable HepG2 cell clones. **(G)** HMGA2 and c-Myc mRNA levels in stable HepG2 cell clones. For **A-G**, all values are presented as mean ± standard error based on at least three independent experiments. *p < 0.05, **p < 0.01, ***p < 0.001. **(H)** The correlation between CCAT1 transcript level and c-Myc mRNA level was measured in 66 HCC tissues. The delta-Ct values (normalized to 18S rRNA) were subjected to Pearson correlation analysis. **(I)** Immunohistochemical scores of CCAT1 in HCC tissues of different CCAT1 expression (Mann–Whitney test, p < 0.001).

To determine whether CCAT1 affects the expression of endogenous let-7 targets, we stably overexpressed CCAT1 WT or CCAT1-Mut in SMMC-7721 cells. The overexpression level of mutant clone is similar to that of WT overexpression clone (Figure 5A). The expression of two known let-7 targets, HMGA2 and c-Myc, were analyzed in CCAT1 stably overexpressed SMMC-7721 cells [28,29]. Overexpression of CCAT1 WT, but not the mutant, increased c-Myc mRNA level (Figure 5B). Although the mRNA level of HMGA2 was not changed, the protein levels of HMGA2 and c-Myc were both upregulated by overexpression of CCAT1 WT, but not the mutant (Figure 5C). We also inhibited let-7 in CCAT1 downregulated HepG2 cells (Figure 5D, E). The inhibition of CCAT1, on the other hand, decreased c-Myc mRNA level, which is abolished by depletion of let-7 (Figure 5F). The protein levels of HMGA2 and c-Myc were decreased by inhibition of CCAT1, which is also abolished by depletion of let-7 (Figure 5G). These data demonstrated that the regulation of HMGA2 and c-Myc by CCAT1 was dependent on the specific binding of let-7. Our results were also consistent with the report that let-7 repressed the translation of HMGA2 mRNA, but did not induce HMGA2 mRNA degradation [30]. We next examined whether CCAT1 is co-expressed with c-Myc and HMGA2 in human HCC samples. We measured the expression levels of CCAT1 and c-Myc mRNA in the same set of 66 HCC tissues shown in Figure 1B. As shown in Figures 5H, CCAT1 transcript level was significantly correlated with c-Myc mRNA level. We also evaluated the immunohistochemical score of HMGA2 protein expression in the same HCC tissues. It was found that the expression level of HMGA2 was significantly higher in CCAT1 high expression group compared to that of CCAT1 low expression group (Figure 5I). All these data suggest an important role of CCAT1 in modulating HMGA2 and c-Myc by competitively binding let-7.

### CCAT1 promotes HCC progression by competitively binding let-7

**Figure 6:**
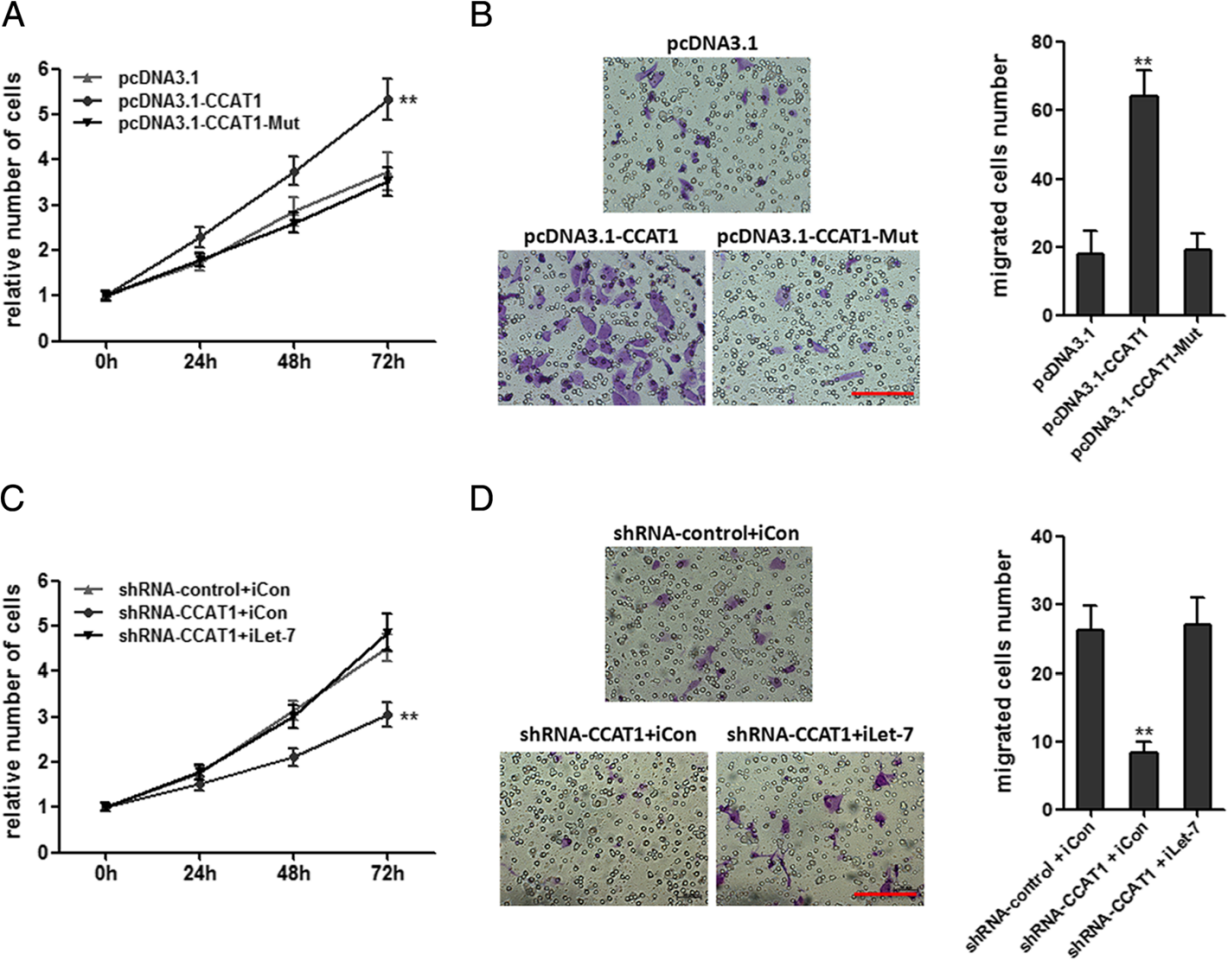
**CCAT1 promotes HCC progression by competitively binding let-7. (A)** The mutation of let-7 binding sites attenuated the promoting proliferation effect of CCAT1 in SMMC-7721 cells. Cell number was determined by the CCK-8 assay, and the relative number of cells to 0 h is presented. **(B)** The mutation of let-7 binding sites attenuated the promoting migration effect of CCAT1 in SMMC-7721 cells by transwell assays. **(C)** Repression of let-7 overcame the inhibitory effects of decreasing CCAT1 on cell proliferation by the CCK-8 assay. **(D)** Repression of let-7 overcame the inhibitory effects of decreasing CCAT1 on cell migration by transwell assays. All values are presented as mean ± standard error based on at least three independent experiments. **p < 0.01, ***p < 0.001.

Functional assays were used to clarify the importance of let-7 binding in promoting HCC progression by CCAT1. CCK-8 assays indicated that the mutation of let-7 binding sites attenuated the promoting proliferation effect of CCAT1 in SMMC-7721 cells (Figure 6A). Transwell assays indicated that the mutation of let-7 binding sites attenuated the promoting migration effect of CCAT1 in SMMC-7721 cells (Figure 6B). Repression of let-7 overcame the inhibitory effects of decreasing CCAT1 on cell proliferation (Figure 6C). Repression of let-7 overcame the inhibitory effects of decreasing CCAT1 on cell migration (Figure 6D). These results showed that CCAT1 promotes HCC progression via competitively binding let-7.

## Discussion

In this study we examined the expression of long noncoding RNA CCAT1 in HCC tissues and pair-matched noncancerous hepatic tissues. Consistent with upregulation of CCAT1 in colon carcinoma and gastric carcinoma tissues from previous reports, our results demonstrate that CCAT1 is also upregulated in HCC tissues in comparison with pair-matched noncancerous hepatic tissues [18,19]. CCAT1 upregulation is correlated with tumor size, microvascular invasion, and AFP. High CCAT1 expression in HCC tissues indicates poor survival of HCC patients. We also identified the function of CCAT1 in HCC cells by applying gain-of-function and loss-of-function approaches. Enhanced expression of CCAT1 promotes the proliferation and migration of HCC cells, while inhibition of CCAT1 inhibits the proliferation and migration of HCC cells. Therefore, our results indicated that CCAT1 functions as an oncogene in HCC.

Several RNA transcripts, including mRNAs and lncRNAs have been identified as competing endogenous RNAs (ceRNAs), such as PTENP1, Linc-MD1, HMGA2, and lncRNA-ATB [14,31-33]. They function as sponges for common miRNAs and abolished the endogenous suppressive effect of these miRNAs on their bona fide targeted transcripts. In this study we demonstrated that CCAT1 functions as a molecular sponge for let-7, upregulates expression of its endogenous targets HMGA2 and c-Myc, and inhibits its function. Our results also found that CCAT1 transcript level was significantly correlated with c-Myc mRNA level and HMGA2 protein level in HCC tissues. Other reports have shown that c-Myc directly bound the promoter region of *CCAT1*, and promoted CCAT1 transcription in gastric carcinoma and colon cancer [18,34]. The effect of c-Myc on CCAT1 in HCC should be further investigated. These showed that c-Myc and CCAT1 maybe upregulated each other, and formed a double positive feedback loop to enhance the robustness of gene networks. Let-7 family miRNAs have been reported to be downregulated in human HCC tissues in comparison with normal hepatic tissues [35]. It is well recognized that let-7 play pivotal roles in tumor by inhibiting proliferation and migration, and inducing apoptosis [26,27]. Our results demonstrated that through competitively binding let-7, CCAT1 further inhibits the function of let-7, which is promoting proliferation and migration of HCC cells. The effects of CCAT1 on proliferation and migration are abolished by the mutation of let-7 binding sites. The inhibitory effects of depletion of CCAT1 on proliferation and migration are overcome by inhibition of let-7. These data support that the role of CCAT1 is dependent upon its binding to let-7. Recently, H19 and HMGA2 have been reported to function as ceRNAs for let-7 miRNA family in different tissues [30,32]. The interactions between these ceRNAs using let-7 microRNA response elements in different cell types require further investigation.

## Conclusions

Collectively, our studies indicate that CCAT1, which is a prognostic factor for HCC, promotes HCC progression by functioning as let-7 sponge. These findings indicate that CCAT1 is an important molecular marker for predicting prognosis, and an important target for HCC therapy.

## Additional file

Additional file 1: Table S1. The predicted miRNA target sites in CCAT1.

## Abbreviations

lncRNA: Long noncoding RNA
HCC: Hepatocellular carcinoma
miRNA: microRNA
ceRNA: Competing endogenous RNA
RIP: RNA immunoprecipitation
